# The Contributory Role of Angiotensin Receptor-Like 1 Gene Multiple Polymorphisms in Hypertension among Northeastern Han Chinese

**DOI:** 10.1371/journal.pone.0086095

**Published:** 2014-01-20

**Authors:** Ruoshan Liu, Hongye Zhao, Yuefei Wang, Yanli Wang, Changzhu Lu, Yu Xiao, Nan Jia, Bin Wang, Wenquan Niu

**Affiliations:** 1 Department of Anesthesiology, Cancer Hospital and Institute, Chinese Academy of Medical Sciences & Peking Union Medical College, Beijing, China; 2 Department of Physiology, Qiqihar Medical University, Qiqihar, Heilongjiang, China; 3 Department of Cardiology, The Second Affiliated Hospital of Qiqihar Medical University, Qiqihar, Heilongjiang, China; 4 Department of Hypertension, Ruijin Hospital, School of Medicine, Shanghai Jiao Tong University, Shanghai, China; 5 State Key Laboratory of Medical Genomics, Ruijin Hospital, School of Medicine, Shanghai Jiao Tong University, Shanghai, China; National Cancer Center, Japan

## Abstract

**Background and Objective:**

Via direct sequencing, we have recently identified six common polymorphisms in angiotensin receptor-like 1 (*AGTRL1*) gene, and found only two polymorphisms were significantly associated with hypertension in a family-based analysis on 1,015 southern Han Chinese. Extending our previous work and considering the ubiquity of epistasis in determining disease susceptibility, we, in this study, sought to explore the potential interaction of *AGTRL1* gene six polymorphisms with hypertension in a large northeastern Han Chinese population.

**Methods and Results:**

This was a case-control study involving 1,009 sporadic hypertensive patients and 756 normotensive controls. Data were analyzed by Haplo.Stats and multifactor dimensionality reduction (MDR) softwares. There were no deviations from Hardy-Weinberg equilibrium for all polymorphisms. The genotypes and alleles of rs7119675 and rs11544374 differed significantly between the two groups (P<0.0005), even after the Bonferroni correction. Under three genetic models, significant association was consistently observed for rs7119675 and rs11544374, and this association was independent of confounding factors. Taking rs7119375 as an example, the odds of having hypertension was 2.46 (95% confidence interval (95% CI): 2.06–2.94), 2.82 (95% CI: 2.29–3.46) and 3.97 (95% CI: 2.37–6.64) under additive, dominant and recessive models (P<0.001), respectively, whereas the adjusted risk estimates were slightly attenuated but still significant. The frequencies of most derived haplotypes differed significantly between patients and controls. Haplotype-phenotype analyses indicated marginal association for triglyceride (P_Sim_ = 0.011) and total cholesterol (P_Sim_ = 0.025) in patients and for triglyceride in controls (P_Sim_ = 0.023). The overall best MDR model included rs11544374, rs7119375 and rs948847 with the maximal testing accuracy of 0.737 and cross-validation consistency of 10 out of 10 (P<0.0001). Further interaction entropy graph suggested that the interaction of rs7119375 with rs11544374 and rs948847 was strongly antagonized.

**Conclusions:**

Our findings demonstrate that *AGTRL1* genetic polymorphisms might contribute to the development of hypertension independently and/or through complex interaction.

## Introduction

Angiotensin receptor-like 1 (AGTRL1) is a 7-transmembrane domain G-protein-coupled receptor, and together with its ligand apelin constitute the promising apelin/AGTRL1 system [1], [2]. Both human and animal studies have implicated the involvement of apelin/AGTRL1 system in regulating body fluid homeostasis and cardiovascular functions [3]–[5]. AGTRL1 and apelin are highly expressed in cardiovascular system, and they are believed to play an important role in counter-regulating the effect of renin-angiotensin system, a classical pathway leading to hypertension. It is therefore reasonable to speculate that *AGTRL1* may be a logical candidate gene in the pathogenesis of hypertension.

The gene encoding *AGTRL1* has two exons, and is mapped on chromosome 11q12.1. Via direct sequencing, we have recently identified six common polymorphisms in *AGTRL1* gene, and in a family-based analysis on 1015 southern Han Chinese we found only two polymorphisms exhibited significant association with hypertension, obesity, and onset age of hypertension [6]. We further confirmed the predictive value of the two significant polymorphisms for the risk of hypertension [7] and coronary artery disease [8] in two case-control studies of southern Han Chinese. However, a literature search revealed little additional evidence for the relationship between *AGTRL1* genetic polymorphisms and hypertension [9], [10]. Considering the ubiquitous nature of epistasis in genetic architecture of common human diseases [11] and to compensate for the inadequacy of our family study [6], we therefore designed a case-control study in a large northeastern Han Chinese population, and sought to explore the potential interaction of *AGTRL1* gene six sequencing-derived common polymorphisms (rs7119375, rs10501367, rs9943582, rs11544374, rs948847 and rs2282623) in susceptibility to hypertension. Besides the study design, what is apparently different from our previous study [6] is this population of northeast China is characterized by genetic homogeneity and geographic stability, and the study subjects are most likely uniform in their environmental exposures, including the habitual intake of high-salt and high-fat diets, and lower rates of hypertension recognition and treatment, all these characteristics making this population more suitable for studying the genetics of hypertension.

## Methods

### Study Population

This was a hospital-based case-control study involving a total of 1765 unrelated subjects as recently reported [12], [13]. All study subjects were Han Chinese and local residents of at least three generations in Qiqihar city, Heilongjiang province in the northeast of China. According to clinical and laboratory examinations, they were classified into two groups: hypertensive patients and normotensive controls. Patients were excluded if they had clinical manifest of secondary hypertension and renal diseases. This study was approved by the institutional review board of Qiqihar Medical University, and was conducted according to the guidelines of Declaration of Helsinki. Informed written consent was obtained from each subject.

Essential hypertension, accounting for more than 90% of all cases of hypertension, was diagnosed as mean systolic blood pressure more than 140 mmHg, or diastolic blood pressure more than 90 mmHg, or the current use of antihypertensive medications. Blood pressure was measured using a calibrated mercury sphygmomanometer with an appropriate adult cuff size by certified examiners. As recommended by Tobin et al [14], for subjects under antihypertensive treatment, blood pressure was corrected by adding 15 mmHg and 10 mmHg to systolic and diastolic blood pressure, respectively, and this method was adopted by Newton-Cheh et al in a genome-wide association study of blood pressure [15].

In total, there were 1009 sporadic patients (males: 54.31%) of age 64.48 (standard deviation: 8.53) years in hypertension group, and the remaining subjects (n = 756), who had normal blood pressure, formed the age- and gender-matched control group.

### Demographic and Clinical Measurement

Age, gender, body weight and height were recorded at enrollment. Body mass index (BMI) was calculated as weight (in kilometers) divided by height (in meters) squared. Fasting venous blood samples were collected. The plasma triglyceride, total cholesterol (TC), high-density lipoprotein cholesterol (HDL-C), blood urea nitrogen (BUN), creatinine, and urea acid were determined enzymatically using available kits and auto analyzers. High-sensitivity C-reactive protein (hsCRP) was measured using a particle-enhanced immunoturbidimetric method (Sangon Biotech, Shanghai, China).

### Genotyping

Genomic DNA was isolated from peripheral blood leukocytes according to a standard phenol-chloroform method, and was stored at −40°C until required for batch genotyping. The genomic organization of *AGTRL1* gene and the localization of six examined polymorphisms are presented in Supplementary Figure S1. Three polymorphisms, rs7119375, rs10501367 and rs9943582, are located in promoter region; two synonymous polymorphisms, rs11544374 and rs948847, are located in exon 1; one polymorphism rs2282623 is located in intron 1. The genotypes of six examined polymorphisms in *AGTRL1* gene were determined by the polymerase chain reaction-ligase detection reaction (PCR-LDR) method as previously described [16].

The PCR reactions were conducted in an EDC-810 Amplifier. The cycling parameters were as follows: 94°C for 2 min; 35 cycles of 94°C for 20 s, 60°C for 20 s, and 72°C for 20 s; and a final extension step at 72°C for 3 min. For each polymorphism, two specific probes were synthesized to discriminate specific bases, and additionally one common probe was synthesized and labeled at the 3′ end with 6-carboxy-fluorescein (FAM) and phosphorylated at the 5′ end. The multiplex ligation reaction was conducted in a reaction volume of 10 µl containing 2 µl of PCR product, 1 µl of 10×Taq DNA ligase buffer, 1 µM of each discriminating probe, and 5 U of Taq DNA ligase. The ligation parameters were 30 cycles of 94°C for 30 s and 56°C for 3 min. After the reaction, 1 µl of LDR reaction product was mixed with 1 µl of ROX passive reference and 1 µl of loading buffer before being denatured at 95°C for 3 min and chilled rapidly in ice water. The fluorescent products of the LDR were differentiated using ABI 3730XL sequencer (Applied Biosystems, California, USA).

The accuracy of PCR-LDR method was validated by randomly selecting 96 samples as internal references in batch genotyping, and the results were exactly the same between repeated measures.

### Statistical Analysis

Demographic indexes and clinical biomarkers were compared between hypertensive patients and normotensive controls by the unpaired t-test and the χ^2^ test when appropriate. Hardy-Weinberg equilibrium was tested by a goodness-of-fit test.

Before and after adjusting for confounding factors including age, gender and BMI, each examined polymorphism was regressed in a binary Logistic model under three genetic models of inheritance, that is, additive model (major homozygotes versus heterozygotes versus minor homozygotes), dominant model (major homozygotes versus heterozygotes plus minor homozygotes), and recessive model (major homozygotes plus heterozygotes versus minor homozygotes). Risk estimates were expressed as odds ratio (OR) and its 95% confidence interval (95% CI). Data were analyzed by STATA software (version 11.0) for Windows (StataCorp LP, College Station, TX, USA). Study power was estimated by the Power and Sample Size Calculations (PS) software (version 3.0.7).

Haplotype frequencies of *AGTRL1* gene six examined polymorphisms between the two groups were estimated by the haplo.em program, and this program computes the maximum likelihood estimates of haplotype probabilities using the progressive insertion algorithm that progressively inserts batches of loci into haplotypes of growing lengths. To avoid chance findings, only haplotype with estimated frequency of at least 3% was considered in this study. Simulated P values were calculated based on 1000 replicates. In addition, the haplo.score program was used to model an individual’s phenotype as a function of each inferred haplotype, weighted by their estimated probability, to account for haplotype ambiguity, and this program is based on score statistics, which provide both global tests and haplotype-specific tests [17]. Both haplo.em and haplo.score programs are implemented in Haplo.Stats software (version 1.4.0), which is operated in the R language (version 2.14, available at the website http://www.r-project.org).

The open-source data-mining multifactor dimensionality reduction (MDR) approach [18], [19] (version 3.0, available at the website http://www.epistasis.org) was employed to identify and characterize the potential interaction of *AGTRL1* gene multiple polymorphisms. This approach aims to construct all possible combinations of six examined polymorphisms, and choose each best model. The accuracy of each model was evaluated by a Bayes classifier in the context of 10-fold cross-validation. In general, a single best model has the maximal testing accuracy and cross-validation consistency. The cross-validation consistency is a measure of the number of times of 10 divisions of the dataset that the best model is extracted. Statistical significance was evaluated using a 1000-fold permutation test to compare observed testing accuracies with those expected under the null hypothesis of null association. Permutation testing corrects for multiple testing by repeating the entire analyses on 1000 datasets that are consistent with the null hypothesis.

Finally, interaction entropy graph was depicted to quantify the synergistic and non-synergistic interaction among polymorphisms. This graph is embedded in MDR software (version 3.0), and information gain value expressed as percentages in the nodes signifies the independent main effect of each polymorphism. The positive and negative information gain values (percentages) on the connected lines indicate synergistic interaction (the red or the orange line) and redundancy or lack of interaction (the green or the blue line) between the polymorphisms, respectively with the zero value indicating independence (the yellow line).

## Results

### Baseline Characteristics

Comparisons of demographic and clinical data between hypertensive patients and normotensive controls are presented in Table 1. There were no significant differences in age (P = 0.751) and gender (P = 0.843) between the two groups. In contrast, BMI (P<0.001), systolic and diastolic blood pressure (both P<0.001), fasting glucose (P<0.001), triglyceride (P = 0.024), and creatinine (P = 0.002) were significantly higher (P<0.05), yet HDL-C was significantly lower (P<0.001), in patients than controls.

**Table 1:** The characteristics of the study population.

Characteristics	Patients (n = 1009)	Controls (n = 756)	P*
Age (years)	64.48±8.53	64.23±10.13	0.751
Gender (males, %)	54.31	53.84	0.843
BMI (kg/m^2^)	27.89±6.29	23.18±3.77	<0.001
Antihypertensive treatment (%)	14.17%	0%	<0.001
Adjusted SBP (mmHg)	147.31±16.52	109.76±17.97	<0.001
Adjusted DBP (mmHg)	89.09±15.92	71.37±11.43	<0.001
Fasting glucose (mmol/L)	6.14±2.15	5.33±1.12	<0.001
TG (mmol/L)	1.90±1.04	1.77±0.95	0.024
TC (mmol/L)	4.59±1.18	4.59±0.91	0.995
HDL-C (mmol/L)	1.12±0.32	1.24±0.34	<0.001
BUN (mmol/L)	5.92±3.88	5.79±4.35	0.557
Creatinine (µmol/L)	87.47±36.79	81.80±25.13	0.002
Uric acid (µmol/L)	329.12±100.31	333.98±95.54	0.374
hsCRP (mmol/L)	12.37±41.42	2.21±3.71	0.001

*Abbreviations:* BMI, body mass index; SBP, systolic blood pressure, DBP, diastolic blood pressure; TC, total cholesterol; TG, triglyceride; HDL-C, high-density lipoprotein cholesterol; BUN, blood urea nitrogen; hsCRP, high sensitivity C-reactive protein.

P values were computed by the unpaired t-test or the Mann-Whitney U test for quantitative variables and by the χ^2^ test for qualitative variables.

### Single-polymorphism Analyses

No deviation from Hardy-Weinberg equilibrium was seen for any polymorphism in both hypertensive patients and normotensive controls. As shown in Table 2, the genotypes and alleles of rs7119675 and rs11544374 differed significantly between the two groups (P<0.0005), even after the Bonferroni correction (Bonferroni significance threshold P = 0.05 divided by the total number of examined polymorphisms (n = 6): P = 0.008). The power to reject the null hypothesis of no differences in the mutant allele frequencies of rs7119675 and rs11544374 between the two groups was both 100%. In contrast, marginal significance was observed for the alleles of rs948847 (P = 0.037), whereas this significance failed to survive the Bonferroni correction.

**Table 2:** Genotype distributions and allele frequencies of *AGTRL1* gene six examined polymorphisms between hypertensive patients and normotensive controls.

Polymorphism*		Patients (n = 1009)	Controls (n = 756)	χ^2^	P**
**rs7119375**	GG	517	565		
	GA	403	173	107.01	<0.0005
	AA	89	18		
	A (%)	28.79	13.82	111.48	<0.0005
**rs10501367**	GG	588	436		
	GA	361	267	0.82	0.665
	AA	60	53		
	A (%)	23.84	24.67	0.33	0.567
**rs9943582**	AA	647	466		
	AG	319	245	2.99	0.225
	GG	43	45		
	G (%)	20.07	22.16	2.27	0.132
**rs11544374**	GG	548	637		
	GA	381	110	180.34	<0.0005
	AA	80	9		
	A (%)	26.81	8.47	189.34	<0.0005
**rs948847**	TT	316	268		
	TG	493	360	4.31	0.116
	GG	200	128		
	G (%)	44.25	40.74	4.35	0.037
**rs2282623**	CC	339	257		
	CT	490	368	0.09	0.958
	TT	180	131		
	T (%)	42.12	41.67	0.07	0.787

Genotypes of six examined polymorphisms were expressed as count (percentage).

P values were computed by using the χ^2^ test based on the 3×2 contingency tables for genotype comparisons and on the 2×2 contingency tables for allele comparisons.

The risk prediction of *AGTRL1* gene six examined polymorphisms for hypertension was undertaken under three genetic models (Table 3). Significant association was consistently observed for rs7119675 and rs11544374 across all genetic models, especially under the recessive model, and this association was independent of confounding factors including age, gender and BMI. Taking rs7119375 as an example, the odds of having hypertension was respectively 2.46 (95% CI: 2.06–2.94), 2.82 (95% CI: 2.29–3.46) and 3.97 (95% CI: 2.37–6.64) under additive, dominant and recessive models (P<0.001 for all), whereas the adjusted risk estimates were slightly attenuated but still significant. For rs948847, there was marginal significance under only additive model (OR = 1.15; 95% CI: 1.01–1.36; P = 0.038), whereas this association was weakened to a nonsignificant level after adjusting for confounders (OR = 1.06; 95% CI: 0.92–1.24; P = 0.417).

**Table 3:** Individual risk prediction of *AGTRL1* gene six examined polymorphisms for the development of hypertension under three genetic models.

Polymorphism- (wild/mutant allele)	Adjust*	Additive model(mm vs. Wm vs. WW)	Dominant model(mm plus Wm vs. WW)	Recessive model(mm vs. Wm plus WW)
**rs7119375-G/A**	No	2.46; 2.06–2.94; <0.001	2.82; 2.29–3.46; <0.001	3.97; 2.37–6.64; <0.001
	Yes	2.11; 1.74–2.56; <0.001	2.37; 1.88–2.97; <0.001	3.26; 1.89–5.63; <0.001
**rs10501367-G/A**	No	0.96; 0.82–1.12; 0.573	0.98; 0.81–1.18; 0.799	0.84; 0.57–1.23; 0.367
	Yes	0.91; 0.77–1.08; 0.279	0.89; 0.72–1.1; 0.279	0.89; 0.58–1.37; 0.59
**rs9943582-A/G**	No	0.89; 0.75–1.04; 0.139	0.9; 0.74–1.09; 0.285	0.7; 0.46–1.08; 0.108
	Yes	0.85; 0.71–1.02; 0.081	0.84; 0.67–1.05; 0.118	0.74; 0.46–1.19; 0.213
**rs11544374-G/A**	No	3.78; 3.06–4.67; <0.001	4.5; 3.57–5.68; <0.001	7.15; 3.56–14.33; <0.001
	Yes	4.31; 3.4–5.47; <0.001	5.32; 4.08–6.94; <0.001	8.93; 4.28–18.59; <0.001
**rs948847-T/G**	No	1.15; 1.01–1.32; 0.038	1.2; 0.99–1.47; 0.068	1.21; 0.95–1.55; 0.123
	Yes	1.06; 0.92–1.24; 0.417	1.15; 0.92–1.44; 0.23	1.0; 0.76–1.32; 0.989
**rs2282623-C/T**	No	1.02; 0.89–1.17; 0.787	1.02; 0.83–1.24; 0.861	1.04; 0.81–1.33; 0.78
	Yes	1.09; 0.93–1.26; 0.28	1.11; 0.89–1.39; 0.352	1.12; 0.85–1.48; 0.407

*Abbreviations:* m, the mutant allele; W, the wild allele. Data were expressed as odds ratio; 95% confidence interval; P for three genetic models of inheritance.

Risk estimates were calculated with or without adjusting for age, gender and body mass index.

### Haplotype Analyses

The frequencies of derived haplotypes (≥3%) from *AGTRL1* gene six examined polymorphisms are presented in Table 4. In total subjects, G-G-A-G-T-T (alleles in order of rs7119375, rs10501367, rs9943582, rs11544374, rs948847 and rs2282623) was the most common haplotype, and was overrepresented in normotensive controls (19.76% versus 6.62% in hypertensive patients, P<0.0005). Besides, the frequencies of haplotypes G-G-A-G-G-T, G-G-A-G-T-C and G-A-G-G-T-C were strikingly higher in normotensive controls than hypertensive patients (P<0.0005), even after the Bonferroni correction (Bonferroni significance threshold P = 0.05 divided by the total number of derived haplotypes (n = 13): P = 0.0038). In contrast, the frequencies of haplotypes G-G-A-A-T-T, G-G-A-A-T-C, A-G-A-G-G-T, A-G-A-G-G-C, A-G-A-G-T-T, A-G-A-G-T-C and G-A-G-A-T-C were significantly higher in patients than controls after the Bonferroni correction. For all significant haplotypes (P<0.0038), the power to reject the null hypothesis of no differences in the estimated haplotype frequencies between the two groups reached 100%, except for two haplotypes A-G-A-G-G-C (91.4%) and A-G-A-G-T-T (55.1%).

**Table 4:** Frequencies of derived haplotypes (≥3% in total subjects) from six examined polymorphisms in *AGTRL1* gene between hypertensive patients and normotensive controls.

Haplotype (%)*	Total (n = 1765)	Patients (n = 1009)	Controls (n = 756)	Simulated P
G-G-A-G-T-T	12.45	6.62	19.76	<0.0005
G-G-A-G-G-T	11.35	10.43	12.58	0.004
G-G-A-G-T-C	9.14	5.11	14.69	<0.0005
G-G-A-G-G-C	8.67	8.92	8.68	0.134
G-G-A-A-T-T	8.10	12.70	2.13	<0.0005
G-A-G-G-G-C	5.78	5.06	6.55	0.006
G-G-A-A-T-C	5.72	8.62	1.36	<0.0005
A-G-A-G-G-T	5.48	7.11	2.69	<0.0005
G-A-G-G-T-C	5.32	3.07	8.15	<0.0005
A-G-A-G-G-C	4.79	5.72	3.36	<0.0005
A-G-A-G-T-T	3.60	4.53	3.15	0.001
A-G-A-G-T-C	2.60	3.78	1.00	<0.0005
G-A-G-A-T-C	2.51	3.68	0.97	<0.0005

Alleles incorporated in a haplotype were in order of rs7119375, rs10501367, rs9943582, rs11544374, rs948847 and rs2282623 in *AGTRL1* gene.

### Haplotype-phenotype Analyses

Global testing of derived haplotypes in their entirety with anthropometric indexes and clinical biomarkers are shown in Table 5. There was marginal omnibus association with triglyceride (global simulated P = 0.011) and TC (global simulated P = 0.025) in hypertensive patients, and with triglyceride in normotensive controls (global simulated P = 0.023).

**Table 5:** Global testing of all derived haplotypes with anthropometric indexes and clinical biomarkers in both hypertensive patients and normotensive controls.

Characteristics	Patients		Controls	
	Global-stat	Global P_Sim_	Global-stat	Global P_Sim_
Age	33.24	0.26	42.72	0.141
Gender	25.95	0.512	41.18	0.125
BMI	43.22	0.105	33.66	0.301
Adjusted SBP	19.26	0.778	39.26	0.227
Adjusted DBP	29.67	0.388	−26.54	0.968
Triglyceride	137.57	0.011	95.26	0.023
TC	63.39	0.025	32.77	0.342
HDL-C	45.96	0.07	48.28	0.106
Glucose	7.22	0.816	26.29	0.154
BUN	−2.19	0.979	37.56	0.068
Creatinine	13.3	0.634	45.68	0.061
Uric acid	39.2	0.133	27.88	0.534
hsCRP	18.74	0.251	15.87	0.362

*Abbreviations:* BMI, body mass index; SBP, systolic blood pressure, DBP, diastolic blood pressure; TC, total cholesterol; HDL-C, high-density lipoprotein cholesterol; BUN, blood urea nitrogen; hsCRP, high sensitivity C-reactive protein.

### Interaction Analyses

To yield more information, we employed a promising data-mining analytical approach MDR to identify and characterize the interaction of multiple polymorphisms in *AGTRL1* gene (Table 6). Each best model is evaluated by the testing accuracy, the cross-validation consistency and the significance level. All best models from one-locus to six-locus were significant at P<0.0001, and had the maximal cross-validation consistency 10 out of 10 except for the four-locus best model. However, the highest testing accuracy was observed for the three-locus best model including rs11544374, rs7119375 and rs948847 (testing accuracy: 0.737).

**Table 6:** Summary of MDR analysis.

Best combination of each model	Testing accuracy	Cross-validation consistency	P
rs11544374	0.6497	10	<0.0001
rs11544374, rs7119375	0.7189	10	<0.0001
rs11544374, rs7119375, rs948847	0.737	10	<0.0001*
rs11544374, rs7119375, rs948847, rs10501367	0.7332	9	<0.0001
rs11544374, rs7119375, rs948847, rs10501367, rs9943582	0.7318	10	<0.0001
rs11544374, rs7119375, rs948847, rs10501367, rs9943582, rs2282623	0.7047	10	<0.0001

The overall best MDR model.

**Figure 1:**
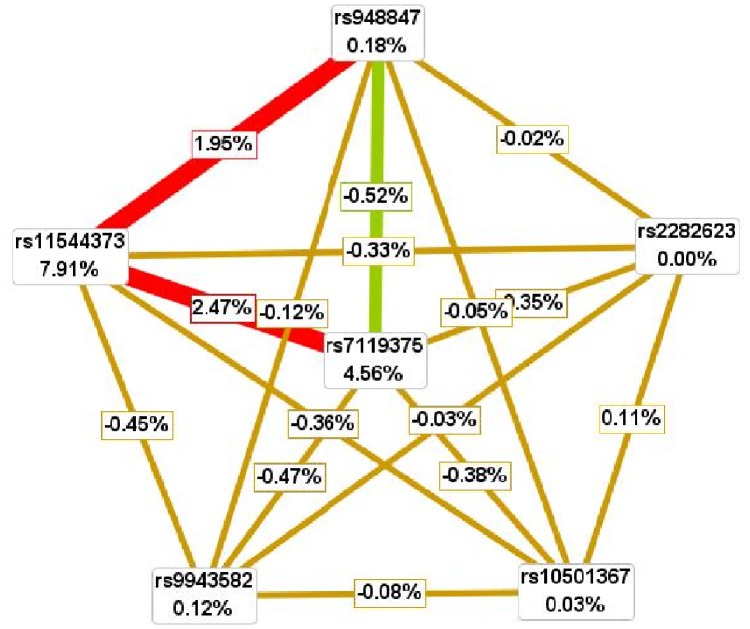
Interaction entropy graph of *AGTRL1* gene six examined polymorphisms.

The interaction entropy graph of *AGTRL1* gene six examined polymorphism is depicted in Figure 1 according to the entropy measures. The two largest independent main effects were observed for rs11544374 and rs7119375 with information gain values of 7.91% and 4.56%, respectively. However, the interaction between rs11544374 and rs7119375 was antagonized with information gain value of 2.47%. What is more, the interaction between rs948847 and rs7119375 was totally inversed with information gain value of −0.33%. In contrast, although the independent main effect was small for rs948847 (information gain value: 0.18%), its interaction with rs11544374 had information gain value of 1.95%.

## Discussion

The present study is an extension of our previous work on the exploration of potential interaction of multiple polymorphisms in *AGTRL1* gene associated with hypertension in a large northeastern Han Chinese population. The most noteworthy finding of this study was that *AGTRL1* genetic polymorphisms might contribute to the development of hypertension independently and/or through complex interaction. The present results not only deepen our understanding of the complex genetic architecture of hypertension, but also provide supporting evidence for the functional role of AGTRL1 in the pathogenesis of hypertension.

Although global scientists have conducted exhaustive research regarding the functional aspects of apelin/AGTRL1 system [20]–[22], data are relatively sparse on the association of multiple genetic defects in this system with hypertension and related diseases in current literature [7], [8], [10]. Considering the close mechanistic relationship between apelin/AGTRL1 system and the renin-angiotensin system [23], it is logical to make *AGTRL1* gene as a candidate gene for hypertension. Accordingly, we have recently sequenced the genomes of *AGTRL1* gene, and identified two of the six common polymorphisms were significantly associated with hypertension in a family-based study involving southern Han Chinese [6]. A question thus arises as to whether the significant association observed in southern Chinese can be extrapolated to northern Chinese, considering the fact that China has a vast territory with obvious regional differentiation in socio-economic development levels and the physical environmental conditions, which can result in different causal factors of disease [24]. For example, it is widely known that high-salt diet can induce or aggravate hypertension in both animal models and humans [25], [26]. However, salt consumption between northern and southern Chinese differs remarkably. As discovered in a national multi-center report, the average urinary sodium excretion in northern Chinese (271 mmol/d) nearly doubled the amount of that in southern Chinese (139 mmol/d), leading to an average 7.4 and 6.9 mmHg increase in systolic and diastolic blood pressure, respectively [27]. Moreover, in our previous family-based analysis on southern Han Chinese, *AGTRL1* gene rs10501367 was found to be associated with the significant risk of hypertension [6], whereas this polymorphism showed no association in northeastern Han Chinese. Instead, we have identified another exonic polymorphism rs11544374 in significant association with hypertension. In view of these divergences, it is encouraging to revisit the contribution of polymorphisms in *AGTRL1* gene to the determination of hypertension in northern Chinese. To the authors’ knowledge, this is the pilot study exploring the genetic susceptibility of *AGTRL1* gene to hypertension in northeastern Han Chinese.

It is worthwhile to note that in our single-locus analyses, *AGTRL1* gene rs7119675 and rs11544374 polymorphisms by itself played an independent leading role in the development of hypertension, since adjustment for confounding factors exerted no substantial impact on the risk estimates. Moreover, this leading role was also reflected in our interaction entropy graph analyses as the two largest information gain values were identified for rs7119675 and rs11544374 polymorphisms. Although residual confounding by incompletely measured or unmeasured physiologic covariates might exist [8], it seems unlikely that our results could be explained by confounding in light of the magnitude of associated significance.

Another important finding in this study of northeastern Han Chinese, which extended our previous work, revealed strong evidence that multiple polymorphisms in *AGTRL1* gene might act interactively in susceptibility to hypertension. It is generally believed that the effect of any single genetic locus will likely be dependent on other genetic loci and environmental factors [11], [28]. To fully address this issue, we adopted a promising data-mining approach MDR. This approach is nonparametric and model-free in design, and has been successfully applied to detect and characterize high-order gene-gene and gene-environment interaction in studies with relatively small samples [23], [29]. By using MDR approach, we identified an overall best three-locus model including the two aforementioned polymorphisms and another nonsignificant polymorphism rs948847. Despite the leading role played by rs11544374, its contribution was obviously antagonized by interacting with rs7119675 and rs948847. Moreover, this interaction was also indirectly reflected in our haplotype analyses. More common haplotypes (frequency >5%) carrying rs11544374-G allele were mostly overrepresented in normotensive controls, and that carrying rs11544374-A allele were consistently overrepresented in hypertensive patients. However for haplotype A-G-A-G-G-T (alleles in order of rs7119375, rs10501367, rs9943582, rs11544374, rs948847 and rs2282623), its frequency was reversely higher in patients than controls in spite of the presence of rs11544374-G allele. One possible explanation for this counterintuitive phenomenon is the simultaneous presence of the risk alleles of rs7119375-A and rs948847-G in this haplotype, and their co-existence might totally offset the protective effect of rs11544374-G allele. Nevertheless, considering the ubiquity of epistasis in determining susceptibility to human hypertension [30], [31], it is a high priority to examine the interaction of more candidate genes or pathways, which may provide valuable insight into the complex mechanisms behind hypertension.

Interpretation of the present study, however, should consider several limitations. First, the retrospective design of this study has inherent drawbacks, and might have introduced survival-related bias [32]. Second, this study of 1765 subjects may be underpowered to demonstrate small risk effects. Third, only six common polymorphisms were examined in *AGTRL1* gene, and it is highly encouraged to incorporate other polymorphisms, especially the low-penetrance polymorphisms in this gene or other validated hypertension-susceptibility genes, such as renin-angiotensin system genes [33]–[35]. More importantly, characterizing the interaction of multiple polymorphisms from different chromosomes is regarded as another effective way to elucidate final genetic architecture of hypertension. Fourth, data on the types of antihypertensive drugs and the treatment period were not available for us, which precluded further analyses of drug effects on our findings. Fifth, the fact that our study subjects were of northeastern Han Chinese descent limited the generalizability of our findings, calling for further confirmation in other ethnic groups.

In summary, our findings demonstrate that *AGTRL1* genetic polymorphisms might contribute to the development of hypertension independently and/or through complex interaction. Even though, we cannot completely exclude the potential confounding from some anthropometric indexes and clinical biomarkers. As hypertension is a multifactorial complex trait, more emphases should be placed on the identification and characterization of high-order genetic and environmental interaction for early detection of individuals prone to the development of hypertension.

## Supporting Information

Figure S1 Genomic organization of human *AGTRL1* gene and the localization of the six examined polymorphisms.
(TIFF)
